# Evaluation of the Detection of Elder Mistreatment Through Emergency Care Technicians Project Screening Tool

**DOI:** 10.1001/jamanetworkopen.2020.4099

**Published:** 2020-05-07

**Authors:** Brad Cannell, Melvin Livingston, Jason Burnett, Megin Parayil, Jennifer M. Reingle Gonzalez

**Affiliations:** 1 Department of Epidemiology, Human Genetics and Environmental Sciences, University of Texas School of Public Health, Dallas; 2 Department of Behavioral Sciences and Health Education, Rollins School of Public Health, Emory University, Atlanta, Georgia; 3 The Texas Elder Abuse and Mistreatment Institute, Forensic Assessment Center Network–Adult Protective Services Division, UTHealth, Houston; 4 McGovern Medical School, UTHealth, Houston, Texas; 5 Department of Health Promotion and Behavioral Sciences, University of Texas School of Public Health, Dallas

## Abstract

**Importance:**

Elder mistreatment is underdetected and underreported. The more than 800 000 medics providing services in every county in the United States represent an important and underused surveillance system.

**Objective:**

To investigate the association between the Detection of Elder Mistreatment Through Emergency Care Technician (DETECT) screening tool use and the number of medic reports made to Adult Protective Services (APS) over a period of approximately 3 years.

**Design, Setting, and Participants:**

This quality improvement study used a difference in difference in differences design and included adults aged 65 years and older who were reported to Texas APS in the study region (246 cities in Denton, Johnson, and Tarrant Counties) between December 31, 2014, and February 28, 2018.

**Exposures:**

The DETECT screening tool.

**Main Outcomes and Measures:**

Reports to APS.

**Results:**

The mean (SD) age of the 11 178 older adults included in this study was 76 (8) years (range, 65-105 years); there was no reported data on patient sex. A total of 18 080 reports of elder mistreatment were recorded. Medics within the study region reported more cases of elder mistreatment during the implementation of the screening tool (relative risk [RR], 4.14; 95% CI, 3.25-5.27). After adjusting for changes in the number of elder mistreatment reports in the comparison groups (ie, underlying changes in reporting trends), the number of reports to APS increased (RR, 3.03; 95% CI, 2.06-4.46). The occurrence of elder mistreatment was validated in 83% (95% CI, 75%-91%) of the reports investigated by APS during the periods when medics did not have access to the screening tool compared with 82% (95% CI, 77%-87%) during the periods when medics had access to the screening tool, indicating that there were no differences in the proportion of reports that resulted in a validated APS investigation.

**Conclusions and Relevance:**

The findings suggest that incorporating the DETECT screening tool into the routine practices of medics is associated with substantial increases in the frequency with which clinicians report potential cases of elder mistreatment to APS.

## Key Points

**Question:**

Is there an association between the use of the Detection of Elder Mistreatment Through Emergency Care Technicians (DETECT) screening tool and the number of medic reports of potential elder mistreatment to Adult Protective Services?

**Findings:**

In this quality improvement study of data from 18 080 reports of potential elder mistreatment, use of the DETECT tool was associated with a significant increase in mean weekly reports of potential elder mistreatment to Adult Protective Services.

**Meaning:**

The findings suggest that incorporating the DETECT tool into the routine practices of medics is associated with substantial increases in the frequency with which they report potential cases of elder mistreatment to Adult Protective Services.

## Teaser

This quality improvement study investigates the association of the Detection of Elder Mistreatment Through Emergency Care Technician (DETECT) screening tool use and the number of medic reports made to Adult Protective Services during approximately 3 years.

## Introduction

Elder mistreatment is an important and prevalent public health problem.^1,2^ Elder mistreatment may manifest in a combination of forms, including emotional abuse, physical abuse, sexual abuse, financial exploitation, and neglect and abandonment.^1,3^ Furthermore, elder mistreatment is associated with multiple serious consequences, including increased rates of physical injuries, depression, emotional distress, functional decline, emergency department use, hospital admissions, and morbidity and mortality.^4,5,6,7,8^ Despite its importance, elder mistreatment remains difficult to detect and often goes unreported.^1,9,10,11,12^

The most widely cited estimate of the annual prevalence of elder mistreatment is approximately 11% of cognitively intact adults aged at least 60 years.^9^ However, this statistic is based on self-report of elder mistreatment collected using a random digit dial telephone survey, and the authors of that study cautioned that these were “precisely the types of events that are notoriously underreported in this age group.”^9^^(p.296)^ Furthermore, Acierno and colleagues^9^ found that only a small number of the instances of elder mistreatment that were disclosed to their research team were reported to the authorities (between 7.9% and 31.0% depending on elder mistreatment subtype). This trend in underreporting is consistent with another recent study,^10^ which found that the identified incidence of elder mistreatment was 24 times greater than the number of cases reported to social services, police, or other legal authorities. Therefore, effective and efficient tools that help to identify older adults who are living with abuse or neglect to connect them with services are urgently needed.

In response to this need, we conceptualized the Detection of Elder Mistreatment Through Emergency Care Technicians (DETECT) project in conjunction with MedStar Mobile Healthcare (MedStar), a large emergency medical services provider in northern Texas, and Texas Adult Protective Services (APS).^12,13,14^ The immediate goal of DETECT was to develop a screening tool that assisted paramedics and emergency medical technicians (medics) with assessing whether or not to report a case of potential elder mistreatment to APS while in the field providing medical services. We believe that medics are a crucial component of our nation’s public health response to elder mistreatment because of the special access they have to otherwise isolated older adults and their living environments. Older adults are 4 times more likely to use emergency medical services than younger adults.^15^ There are more than 800 000 medics providing emergency medical services in every county in the United States.^16^ By systematically observing older adults in their physical and social environment, medics often have opportunities to observe indicators of elder mistreatment that are not available to other medical professionals.^13,14^

We previously pilot-tested a 26-item version of the DETECT screening tool with medics in the field between September 17 and October 26, 2015.^12^ We found that the DETECT tool was feasibly incorporated into emergency medical services standard operating procedures and used by medics in the field. In addition, we found preliminary evidence that the introduction of the DETECT screening tool was associated with an increased number of cases of potential elder mistreatment being reported to APS by medics. However, this pilot study had many limitations. Among them was the short duration of the study (approximately 5 weeks) and the lack of a comparison group. In the current study, we aimed to investigate changes in the number of medic reports to APS associated with DETECT screening tool use during a period of approximately 3 years using both concurrent and historical controls.

## Methods

### Brief Overview of DETECT Screening Procedures

This quality improvement study used a difference in difference in differences (DDD) design and included adults aged 65 years and older who were reported to Texas APS in the study region (246 cities in Denton, Johnson, and Tarrant Counties) between December 31, 2014, and February 28, 2018. Complete details about the development of the DETECT screening tool, including a rationale for why existing tools were inappropriate for this setting, have been published previously.^12,13,14^ The design and conduct of this study was reviewed and approved by the Committee for the Protection of Human Subjects at the University of Texas Health Science Center at Houston. A waiver of informed consent was granted by the Committee of the Protection of Human Subjects because it was often not feasible to request consent in an emergency medical situation. This study followed the Standards for Quality Improvement Reporting Excellence (SQUIRE) reporting guideline.

In brief, the DETECT tool was designed specifically to help medics identify potential elder mistreatment among community-dwelling older adults during an emergency response. The DETECT tool relies entirely on the medics’ systematic observations of the older adults’ physical and social environment; no direct questioning of the older adult or their caregivers is involved. As previously noted, a 26-item version of the DETECT screening tool was used by MedStar between September 17 and October 26, 2015. On October 27, 2015, MedStar stopped using the DETECT screening tool while we conducted an evaluation of the data collected during that initial pilot period. In that evaluation, we found preliminary evidence of the effectiveness of the DETECT screening tool.^12^

We also found that responses to 12 of 26 items had 0 or near 0 variance.^12^ Therefore, between February 2, 2017, and February 28, 2018, a revised 14-item version of the DETECT screening tool was used by MedStar. This use pattern resulted in 4 distinct study periods: (1) pre-DETECT, which was the period before the initial use of the DETECT screening tool (from December 12, 2014, to September 16, 2015); (2) DETECT pilot study from September 17 to October 26, 2015; (3) wash-out period from October 27, 2015, to February 1, 2017; and (4) DETECT 1-year observational study from February 2, 2017, to February 28, 2018.

In each of the active screening periods (DETECT pilot study and 1-year observational study), the DETECT screening tool was embedded in MedStar’s electronic patient care reporting system. Medics were automatically prompted to complete the DETECT screening tool during an eligible 911 response. An eligible 911 response was defined as a call for a community-dwelling patient who was 65 years or older in the setting of the patient’s residence with the patient residing in the community (eg, private home, unlicensed adult foster home, or unlicensed board and care home). Other types of residences (eg, licensed skilled nursing facilities) were excluded because reports of elder mistreatment in these settings are generally not investigated by APS in Texas.^17^

All 14 screening items are listed in the Box. Medics were instructed to make a report of potential elder mistreatment to APS every time they observed at least 1 of the screening items.

Box. **Detection of Elder Mistreatment Through Emergency Care Technician Screening Tool Items**

Unusual odor (eg, urine, feces)
Inside of the home is in extreme disarray or there is hoarding
Living environment poses a health or safety concern (eg, fire hazard, insect or rodent infestation, or urine or feces present)
If caregiver present, they appear to lack knowledge of the patient or older adult's medical needs
If a caregiver is present, they appear unengaged and inattentive in caring for the patient or older adult
If a caregiver is present, they appear frustrated, tired, angry, or burdened by the patient or older adult
If a caregiver is present, they appear overly concerned (eg, anxious, hovering)
Is the patient or older adult isolated in the home?
Does the patient or older adult appear depressed, anxious, or emotionally distressed for reasons other than their immediate medical condition?
Does the patient or older adult have poor personal hygiene (including soiled in urine or feces)?
Is the patient or older adult inadequately clothed or wearing dirty, torn, or soiled clothing?
Does the patient or older adult have difficulties taking their prescribed medications as directed?
Does the patient or older adult appear to be hoarding or saving old medications?
Does the patient or older adult have unmet needs for assistance with eating, toileting, transferring, dressing, or bathing?

### Study Design

The primary aim of this study was to estimate the association between use of the DETECT screening tool and the likelihood of reporting elder mistreatment to APS. We used data provided by the Texas Department of Family and Protective Services, which included the outcomes of all APS investigations, to conduct this evaluation. The data included all reports of elder abuse, neglect, and exploitation made to APS between December 31, 2014, and February 28, 2018—encompassing all 4 of the study periods outlined above. In addition, the data included reports from Denton, Johnson, and Tarrant Counties in Texas. Together, these counties contain each of the 15 cities where MedStar is provides emergency medical services, as well as the areas immediately surrounding MedStar’s service area.

We used a DDD design to estimate the association between DETECT and both the number of reports of elder mistreatment made each week and the probability of a report being validated by APS. The DDD design provided plausible associations by estimating the change in reporting before and after the implementation of DETECT from medics within the Medstar service area as a function of the change among nonmedics and medics outside the service area.^18^ The DDD design provided 2 levels of design controls. First, it compared the change in reporting among medics in the Medstar service area with changes among medics outside the service area who had not received the DETECT screening tool, accounting for differences in reporting elder mistreatment by medics over time. In addition to this between-service area comparison group, the DDD design compared the change in reporting within the Medstar service area between medics and nonmedics. This additional comparison group controlled for time-dependent confounding specific to the Medstar service area.

### Statistical Analysis

To estimate the change in the number of elder mistreatment reports associated with DETECT, we estimated a negative binomial regression model on aggregate counts of elder mistreatment reports by study week and intervention group. This model included a 3-way interaction between dummy variables for whether the DETECT screening tool was in use (including both the primary DETECT implementation period and the 5-week pilot study in 2015), whether the reports came from within the Medstar service area, and whether the reports came from a medic, as well as all lower-level terms. The regression coefficient for the 3-way interaction estimated the DDD as the estimated change in the number of elder mistreatment reports among medics using DETECT after subtracting the estimated change in elder mistreatment reports among the control groups. To estimate the change in the probability of a report of elder mistreatment being validated in association with DETECT, we analyzed similar logistic regression models at the level of the individual report. To account for nonindependence over time, all models were estimated as generalized estimating equations using proc genmod in SAS, version 9.4 (SAS Institute Inc), clustering the SEs at the week level.

The primary assumption of the DDD design was that the trends in elder mistreatment reporting before DETECT were parallel between medics who received DETECT and the comparisons groups. To test the robustness of our results to the parallel trends assumption, we estimated additional models including group-specific linear time trends. No substantive differences were found between the primary models and these sensitivity analyses.

## Results

Among 11 178 adults included in this study, the mean (SD) age was 76 (8) years (range, 65-105 years); there was no reported data on patient sex. The data included all 18 080 reports of elder mistreatment, neglect, and exploitation received by Texas APS in the time and geographic area described above. Of those reports, 667 (4%) were made by medics.

The number of weekly elder mistreatment reports made to APS were plotted as a black line in Figure. During the first study period (Pre-DETECT), MedStar medics completed a mean of 0.8 reports (95% CI, 0.5-1.1 reports) of elder mistreatment to APS per week. During the 5-week pilot study, the mean number of reports per week increased to 3.0 (95% CI, 1.3-4.7 reports). After removal of the DETECT tool from MedStar’s electronic patient care reporting system (study period 3, wash-out period), mean weekly reporting decreased to 1.6 reports (95% CI, 1.2-2.0 reports). After reintroduction of the DETECT screening tool in study period 4 (1-year observational study), mean weekly reporting increased to 5.7 reports per week (95% CI, 5.1-6.4 reports per week).

**Figure.**
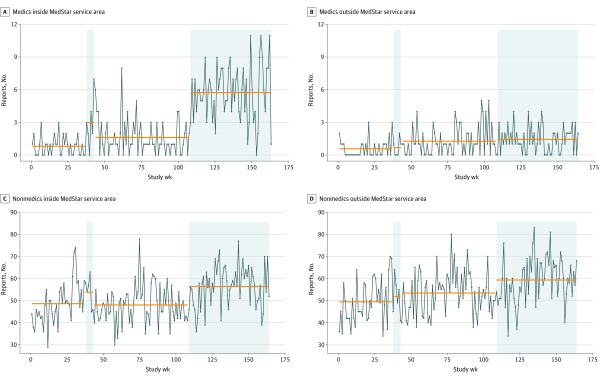
Weekly Reports of Elder Mistreatment to Texas Adult Protective Services by Reporter Type and MedStar Service Area, December 31, 2014, to February 28, 2018 MedStar’s service area included Denton, Johnson, and Tarrant counties, Texas, with 13 cities inside the service area and 246 cities outside the service area. Blue-shaded areas represent periods of DETECT screening tool use including a 5-week pilot study between September 17 and October 27, 2015, and the 1-year observational study that began on February 1, 2017. Orange lines represent the mean number of reports in each period. The variance is captured in the black time series line. Nonmedics include any other person who made a report to Adult Protective Services (eg, physicians, clergy, caregivers, or private citizens) and were included as a function of the difference in difference in differences design. Outcome changes observed in the nonintervention groups could not be an outcome of the DETECT tool, and thus were assumed to represent different potential sources of bias.

The estimated relative change in each group is presented in the Table. Medics within the Medstar service area reported more than 4 times as many cases of elder mistreatment during the implementation of DETECT (relative risk, 4.14; 95% CI, 3.25-5.27). However, we also observed small before-and-after changes among nonmedics inside the MedStar service area and medics outside the MedStar service area, which indicated potential confounding in this crude estimate. After adjusting for changes in the number of elder mistreatment reports in these comparison groups (ie, underlying changes in reporting trends), the DDD estimate indicated an increase in reports (relative risk, 3.03; 95% CI, 2.06-4.46) associated with the DETECT screening tool. The occurrence of elder mistreatment was validated in 83% (95% CI, 75%-91%) of the reports investigated by APS in the periods when MedStar medics did not have access to the DETECT screening tool (study periods 1 and 3) compared with 82% (95% CI, 77%-87%) in the periods when MedStar medics had access to the DETECT screening tool (study periods 2 and 4), indicating that there were no differences in the proportion of reports that resulted in a validated APS investigation. In other words, there was no evidence that the increases in reporting associated with the DETECT tool use were disproportionately invalid or inappropriate reports.

**Table.** Association Between Use of DETECT and Changes in the Number of Elder Mistreatment Reports by Reporter Type and Service Area^a^

Group	Relative risk (95% CI)
Medics inside the MedStar service area	4.14 (3.25-5.27)
Nonmedics inside the MedStar service area	1.16 (1.09-1.22)
Medics outside the MedStar service area	1.32 (0.96-1.82)
Nonmedics outside the MedStar service area	1.12 (1.05-1.19)
DETECT DDD estimate	3.03 (2.06-4.46)

Abbreviations: DDD, difference in difference in differences; DETECT, Detection of Elder Mistreatment Through Emergency Care Technicians.

^a^ Nonmedics include any other person who made a report to Adult Protective Services (eg, physicians, clergy, caregivers, and private citizens) and were included as a function of the DDD design. Outcome changes observed in the nonintervention groups could not be an outcome of the DETECT tool, and thus were assumed to represent different potential sources of bias.

## Discussion

Medics are uniquely positioned to play a vital role in detecting elder mistreatment, especially among older adults with the greatest medical burden who may be isolated and at increased risk for abuse. Tools that make it easier for medics working in the field to identify and report situations in which elder mistreatment and/or neglect is potentially occurring to the appropriate protective services agencies may help initiate earlier intervention to people who would otherwise not have that option. This study provides evidence that incorporating the DETECT screening tool into the routine practices of medics may be associated with substantial increases in the frequency in which they report potential cases of elder mistreatment to APS. Of importance, there was no evidence that the observed increase in reporting frequency was accompanied by a decrease in the appropriateness of those reports (ie, there was no difference in the proportion of cases that were ultimately validated by APS). Use of the DETECT tool was associated with the investigation and intervention of elder mistreatment cases that may have otherwise gone unnoticed and unreported.

Although medical professionals are mandatory reporters of elder mistreatment in every state, purposeful screening is not a common practice.^19^ Long screening forms, lack of standardization among the forms available, lack of knowledge regarding elder mistreatment indicators and appropriate responses, and lack of endorsement for elder mistreatment screening by the US Preventive Services Task Force are all barriers to screening. The DETECT screening tool provides a brief observational tool for medics in which most information is gathered through routine interactions with older adults in their homes. It also provides reporting guidance while delineating that the role of the medic is not case management but detection and reporting. This fits the expectations of a screening tool as described by Fulmer and O’Malley, who suggested that “the best role for screening instruments is to heighten the professional awareness of the possibility of elder mistreatment and alert clinicians to signs and symptoms that might otherwise be missed.”^20^^(p151)^

Although the US Preventive Services Task Force report found no evidence that reporting elder mistreatment causes harm, the report suggested that potential harm could occur.^21^ This is true in any situation in which there is a power imbalance or dependency between an alleged perpetrator and victim. However, use of DETECT was not associated with an increase in the proportion of false positives reported to or investigated by APS and, therefore, was not likely associated with greater increased risk for harm compared with other reports. In addition, despite adequate evidence^21^ that detection and screening are associated with minimization of harmful outcomes such as future abuse and mortality, this finding was likely confounded by the complexities of abuse situations in which the individual experiencing the harm protects the individuals causing the harm, by the lack of field-based consensus on what constitutes a good outcome, and by the lack of rigorously tested interventions in this population. The elder mistreatment field recognizes these challenges, but nonetheless, it is believed that some action is better than no action even if to only reduce abuse and improve quality of life for a short time.

### Limitations

This study has limitations. First, the sample for this analysis was large but geographically limited. Therefore, the results may not generalize well to other geographic areas. Second, at this stage in the DETECT project, our goal was to develop a tool that helps medics comply with state mandatory reporting laws (ie, recognizing and reporting potential elder mistreatment to the authorities). Although this is an important first step, it is different from accurately identifying true elder mistreatment occurrence. The proportion of reports that were validated by APS investigation did not decrease during periods of DETECT tool use; however, there are legitimate reasons to be cautious about using APS investigation outcomes as a criterion standard for true elder mistreatment occurrence.^22^ We are currently gathering more robust outcomes data that we will use to evaluate the concordance between positive DETECT results and true elder mistreatment occurrence in future studies.

## Conclusions

Use of the DETECT tool was associated with the investigation and intervention of elder mistreatment cases that may have otherwise gone unnoticed and unreported. The findings suggest that incorporating the DETECT screening tool into the routine practices of medics is associated with substantial increases in the frequency in which medics report potential cases of elder mistreatment to APS.
